# Some Unusual Features of Lead Poisoning

**Published:** 1909-06-05

**Authors:** Thomas Oliver

**Affiliations:** Physician to the Royal Victoria Infirmary, and Professor of Physiology in the College of Medicine, Newcastle-upon-Tyne


### Hospital Clinics.

### SOME UNUSUAL FEATURES OF LEAD POISONING.

By Sir THOMAS OLIVER, M.D., LL.D., D.Sc.,F.R.C.P.; Physician to the Royal Victoria Infirmary, and Professor of Physiology in the College of Medicine, Newcastle-upon-Tyne.

(A Lecture Delivered at The Polyclinic, May 19, 1909.)

(*Continued from page* 232.)

The peculiarity about plumbism is that lead is not poisonous in a single dose. In order to cause paralysis the poison must be taken into the system in small doses over a long period. Occasionally, where there is idiosyncrasy, paralysis may occur in the course of a few weeks. As causes contributing to the development of paralysis in persons who work among lead must be mentioned alcohol and fatigue. It is important to remember that paralysis may come on a long time after the patient has ceased coming into contact with lead. Opinion is still divided as to whether paralysis in lead poisoning is due to a peripheral or a central lesion. Where a lesion has been found in the anterior cornual cells, it is taught that this may be a sequel to the peripheral neuritis. Most writers regard lead paralysis as the result of a peripheral neuritis, but the paralysis does not always follow in its distribution the branches of a peripheral nerve. The muscles affected act synergetically or in physiological groups without any reference to the distribution of nerve fibres, and while atrophy is usually also present, occasionally there is atrophy alone without noticeable paralysis. The onset of the paralysis may be comparatively rapid or slow. In one patient loss of power in the hands occurred when combing her hair; often the paralysis develops between night and morning, or is more gradual and preceded by a sense of muscular tiredness or of weight, cramp-like sensations or tremors, and, less frequently, by hyperæsthesia or anæsthesia. Occasionally the affected muscles are tender, pain is felt on pressing the affected nerve, or there may be patches of analgesia and anæsthesia, as, for example, over the inner and posterior parts of the forearm in wrist-drop. These are not patches of hysterical anæsthesia, for when the skin is pricked it readily bleeds.

The functional disturbances in peripheral neuritis are motor, sensory, or mixed. In lead paralysis there is little sensory disturbance. It is usually motion and muscular nutrition that are alone affected. In well developed instances of lead paralysis the peripheral nerves frequently show lesions of a gross character, but although few, if any, changes are observed in the cells of the anterior cornua of grey matter of the spinal cord, these cells have in all probability not escaped. The nearer to the periphery the nerves examined, the more pronounced are the signs of interstitial neuritis. It is difficult to say how far up the nerves the changes extend. Sometimes they reach as high up as the brachial plexus and the anterior spinal roots. In only a few cases have pathological changes been observed in the anterior cornual cells. Several of these are atrophied, and there is a multiplication of the nuclei of the neuroglia. Although lead is believed to have a specific action on muscular fibre, inciting unstriped muscular fibre especially to contract, yet it has not been found that the paralysed muscles in lead poisoning contain more lead than the non-paralysed. The loss of power, therefore, cannot be explained on the predilection of certain muscles for lead. On the purely peripheral neuritis theory it is difficult to explain the almost constant freedom of the supinator longus, also the implication of extensors, as opposed to the flexors. My own feeling is that while peripheral lesions predominate, the anterior cornual cells also tend to become involved, and possibly, too, muscular fibre itself; for an isolated muscle exposed to lead is more readily fatigued than a healthy one. Tiredness of muscle is a common complaint of lead workers before paralysis develops.

Occasionally appendicitis is simulated by lead colic and *vice versa*; so too is pelvic disease. The abdomen has been opened by surgeons for supposed appendicitis when the illness was lead colic, and treatment has been adopted for supposed pelvic peritonitis when the malady was plumbism. As in nearly all cases of lead poisoning indican is present in the urine, I regard indicanuria as of some assistance in framing a diagnosis. Formerly I was of the opinion that indicanuria pointed to an excess of intestinal fermentation, but I am convinced that it occurs without intestinal putrefaction. Shown by adding chloroform to equal parts of the urine and hydrochloric acid, it may be due to functional derangement of the liver. As bearing upon this point, it is of interest to remember that during lead colic sulphocyanide of potassium, which gives a reddish brown colour with a weak solution of liq. ferri. perchlor., disappears from the saliva, as the late Dr. Samuel Fenwick first demonstrated, and reappears in it on the cessation of the abdominal pain.

As an illustration of diseased kidneys and arteries with high blood-pressure ending in cerebral hæmorrhage in a lead worker, the following notes of a case of pontine hæmorrhage are worth recording. W. C., aged 56, a furnace man in a red-lead works, was admitted into the Infirmary under my care, complaining of abdominal pain and weakness of his legs. He was extremely anæmic. There was no well-marked blue line on the gums, but a few blue patches on the inside of the lower lip opposite decayed teeth. Pulse 60, of high tension, equivalent to 275 mm. Over the mitral area a prolonged reduplicated first sound could be heard, the second aortic sound was accentuated. Urine, which contained albumen, had a sp. gr. 1,009; 64 ounces were passed daily, containing 300 grains of urea. The red blood cells numbered 2,500,000 per c.mm. and the white corpuscles 6,000, the hæmoglobin stood at 50 per cent. No lead was found in the urine at first, but after a few doses of potassium iodide it appeared. Its presence in the urine was accompanied by extremely severe headache and by an aggravated loss of eyesight not due to a rise in the arterial tension, for under the administration of potass. iodide it fell from 275 to 256, and ultimately to 250 mm. Hg. Mr. Wardale, ophthalmologist to the Royal Victoria Infirmary, on examining the eyes, found optic neuritis with hæmorrhages in both eyes, also small cotton wool-like patches in the left eye. As the potassium iodide had increased the headache the drug was stopped. By degrees the pain lessened and the sight improved. A week or ten days afterwards the patient was seized with convulsions and died. At the autopsy by Dr. George Hall the mitral cusps were found healthy and the aortic cusps thickened. The heart weighed 24½ ounces. The kidneys were contracted. There were marked atheroma of the vessels at the base of the brain and a small aneurysm of the basilar artery. On section of the pons a large hæmorrhage was found. In the right cerebral lobe in front of the internal capsule a hæmorrhage the size of a chestnut was found of older date than that in the pons. On microscopical examination the brain cells in the cortex were found to be healthy, but the arterio-capillaries of the surface were thickened, and capillary nucleation was better marked than usual. In the liver Professor Bedson found considerable traces of lead.

It is known by those who have had the fortune to see autopsies in cases of acute lead encephalopathy how completely absent may be pathognomonic morbid appearances. The details of the following case, which came under the care of my colleague Dr. W. E. Hume, which I saw with him, and for permission to publish which I express my thanks, are therefore of more than ordinary interest: A plumber, aged 35, was admitted into the Infirmary in a state of unconsciousness on November 30, 1908. Three days previously he had returned from work with severe headache, which continued all night, but on the following morning, although feeling ill and dizzy, he returned to work, where shortly afterwards he was seen to fall and to be convulsed. Within an hour he had sufficiently recovered to go home, where, in the evening, he had another convulsion. Previous to this breakdown he had attended the Eye Infirmary for failing vision. On admission under my colleague's care knee-jerks were present on both legs, the right arm and leg were rigid and motionless, while the left arm and leg were moved convulsively. Babinsky's sign was absent on the right side, but present on the left. There was optic neuritis, more marked in the right eye. The temperature was 100° F., rising to 101°. There was vomiting with incontinence of urine and fæces. There was no albuminuria. Nothing abnormal was heard over the cardiac area; blood pressure 105. As the persisting unconsciousness and the physical signs suggested cortical irritation, possibly tumour, Mr. Grey Turner trephined the left side of the skull, but nothing was found except slight œdema of the brain. On the following day the patient died. At the post-mortem examination by Dr. George Hall the internal organs of chest and abdomen were, practically speaking, healthy. On removing the calvarium there was observed slight thickening of the pia-arachnoid membranes over the vertex, but these were not adherent. The convolutions of the right brain, also of the left side in the neighbourhood of the operation area, exhibited a distinctly watery appearance. There was no flattening of the convolutions. Here and there the vessels at the base of the brain showed small patches of atheroma, and small petechial hæmorrhages were seen in the substance of the brain, but no gross hæmorrhage. The cerebellum was normal. At the autopsy, beyond slight œdema of the brain which suggested uræmia or plumbism, there was nothing to explain the cause of death, and no morbid appearances sufficiently marked to make a diagnosis absolute. This was only possible after a chemical analysis of the internal organs. The following is Professor Bedson's report:—

Brain contains 3 parts of lead per million of tissue.
Liver contains 6 parts of lead per million of tissue.
Kidney contains 50 parts of lead per million of tissue.
Spleen contains 40 parts of lead per million of tissue.

Here then is a case in which, but for the chemical analysis of the urine and internal organs, the diagnosis would have remained more or less only a matter of probability. The interesting point in the chemical analysis is the small amount of lead found in the liver compared with the kidneys and spleen. Usually this organ contains the largest amount of lead. The patient's symptoms pointed to brain, and yet a mere trace of lead was found therein after death compared with the kidney.

The interesting paper on lead poisoning in children by Dr. Jefferies Turner,* in which he reminds us of the differences in the symptoms between children and adults, contains, practically speaking, all that can be said of the matter. Dr. Turner tells us that in many of his patients it is not so much a continuous blue line which is present on the gums as a series of extremely small black dots; that gastro-intestinal symptoms are less well defined, and that while vomiting is frequent and persistent, gentle pressure upon the abdomen usually gives relief. Constipation, although frequent, is not invariable. Pains in the legs, usually worse at night, are much complained of. These are followed by "toe drop." Wrist drop is not a characteristic feature of lead poisoning in childhood. The tibialis anticus and the extensor longus digitorum pedis are the earliest muscles to become affected. Turner draws attention to the occurrence of acute diaphragmatic paralysis attended by cardiac weakness, rapid pulse, great depression and vomiting, also to the occurrence of a "localised basic meningitis," in which the children complain of severe headache, with pains in the neck running down the arms, retraction of the head, vomiting, and internal squint. Squint may be the only physical sign present; both external recti are involved, occasionally, too, some of the muscles supplied by the third nerve, the levatores palpebrarum, escaping. When convulsions do not develop usually the signs of meningitis disappear, but imperfect vision may remain for a time. In some instances eyesight is entirely lost owing to neuro-retinitis.

* *British Medical Journal,* April 10, 1909, p. 895.

To this picture of plumbism in children in Queensland, due to accidental poisoning from the white lead with which the balconies of the houses are painted, I feel that I ought to add the still more harrowing accounts of the lead poisoning of children in Hungary, to which attention is drawn by Dr. Adalbert Chyzer,* of Buda Pesth, and in which heredity and exposure to lead play the principal rôle, since the manufacture of cheap pottery in many parts of Hungary is a home industry. The potting shops adjoin the dwelling houses. Dr. Chyzer found that the earth taken from the living-room of the houses contained sometimes as much as 8.7 per cent. of lead, that the blue linen gown and clothes worn by a boy five years old contained 0.243 gramme of lead, and his cap 0.0144 gramme. A workman's clothes contained 3.90 grammes of lead, and a pillow on which a child was lying suffering from plumbism contained 0.0897 gramme of lead. In an infant one year old Chyzer found deposits of lead in the gums and on the teeth. Sometimes it is in the form of a dirty grey line at the neck of the teeth or a violet border upon the gums. The milk teeth of potters' children decay early and become black. There is frequently toothache with complaint of pain in the jaws; the glands underneath and the bone itself often become enlarged. The early shedding of the milk teeth may be attended by a periostitis and by a limited necrosis requiring surgical removal of the dead bone. Chyzer has seen three cases of periostitis and 10 of necrosis. He attributes the necrosis to the action of lead, since in all the little patients and their parents there were signs of subacute lead poisoning. Rickets is frequently seen in the children; most of them are extremely stunted in their growth, others again are subjects of a chronic form of saturnine encephalopathy. A few of them, in addition to paralysis and atrophy of the legs, suffer from blindness and convulsive seizures. Chyzer draws attention to what I have never met, the occurrence of hæmatemesis in adult potters. Among the potters of Austria-Hungary we are brought face to face with some of the worst evils of lead poisoning, for wherever pottery is a home industry many of the children are not only the offspring of parents that are themselves lead poisoned, but they are reared in an atmosphere which cannot but be contaminated with lead dust, and thus we find the children, under these circumstances, the unfortunate victims both of an inherited depraved constitution and of infection. The animals born in or near the houses die. A potter cannot keep a singing bird, nor can he rear fowls, for the dust of the workshop kills them. The cat is extremely susceptible to lead. It soon develops colic, and if the pain persists for long the animal becomes almost mad; it keeps knocking things over in its wild career, or dashing itself against obstacles in its path; it ends by throwing itself into water.

### The Immediate Effects of Lead upon the Heart and Circulation.

Lead when taken in a single dose is not a powerful poison. I have had patients who have intentionally taken one large dose of acetate of lead, and who have quickly recovered without any bad effects. With the view of ascertaining upon which organ or organs lead acts directly when introduced into the system by an intravenous channel, I carried out a series of experiments, with the assistance of my colleague Dr. R. A. Bolam, to whom I beg most heartily to acknowledge my sense of indebtedness. Sollman* reminds us that lead is a specialised poison for many tissues and organs. It is a general poison to protoplasm, and exercises a more injurious influence upon the more highly developed tissues, such as nerve and muscle, than upon the blood corpuscles. In our experiments† upon anæsthetised dogs varying quantities of solutions of nitrate of lead were carried into the veins, means being taken to register the blood pressure. When 5 c.c. of distilled water were injected into a dog under ether, no appreciable effect was produced on the arterial pressure. On the other hand the injection of 5 c.c. of a 1-per-cent. solution of lead nitrate was followed shortly afterwards by a slight fall of the arterial pressure; 5 c.c. of a 2-per-cent. and subsequently a similar quantity of a 5-per-cent. were both followed by a slight fall, which in each instance was fairly quickly recovered from. Ten c.c. of a 10-per-cent. solution was followed by a very marked fall and slow recovery. A second injection of 10 c.c. of a 10-per-cent. solution was succeeded by a great fall of arterial pressure. The declension was rapid, and shortly afterwards the heart ceased beating. Meanwhile respiration continued, and after the lapse of three to four minutes the heart, recovering itself, began to beat again, feebly at first, and then by degrees more strongly until the beats became normal. On injecting 5 c.c. of a 10-per-cent. solution of lead nitrate the arterial pressure again fell, but recovered itself, while 10 c.c. of a 10-per-cent. solution was succeeded by an extremely rapid fall; respiration continued but gradually became interrupted, the heart's beats, becoming feebler and ineffective, ceased, as also did respiration. At the autopsy, 24 hours afterwards, the urine was found to be free from albumen. It contained a reducing substance like sugar, but which was not sugar. The wall of the heart was flabby, especially that of the right ventricle, which contained a small quantity of dark fluid blood. The lungs were healthy, kidneys congested, liver dark but healthy. The abdominal veins were extremely full and tense. The brain, which was congested on the surface, was pale internally.

By a male dog, to whom under chloroform 5 c.c. of a 5-per-cent. solution of lead nitrate was administered intravenously, amyl nitrite was inhaled on the restitution of the blood pressure, the immediate result being, as expected, a renewed fall of pressure. The effect of a fresh injection of nitrate of lead was a more than usual rapid fall of pressure with increased respiration, due apparently to stimulation of the respiratory centre for want of blood. Heart's beat and respiration ceased, but respiratory efforts were renewed at intervals and repeated for

* Des Intoxications par le Plomb se présentant dans la Céramique en Hongrie, 1908.

* *Text-book of Pharmacology*, p. 638.

† See diagrams published May 29, pp. 229 and 231.

three minutes, after which they ceased, the heart not beating at all. At the autopsy there was nothing to note except that the heart was filled with fluid blood and the abdominal veins were turgid.

The effects of the intravenous injection of lead are the same, whether the animal used is a dog or a rabbit. There is always, if the dose is sufficiently large, a fall of blood pressure, and the fall is general. It occurs in the splanchnic area and in the general systemic vessels. In the case of lead there is no immunity produced, as is the case with some other poisons. The blood pressure falls gradually but still fairly rapidly, and the heart ceases to beat. Both may be recovered, and the cardiac sounds again become audible. Lead when introduced directly into the blood acts partly and primarily as a vaso-dilator, but when in lethal doses it seems to have a further direct action upon the heart, as the tracings show. Respiration is apparently not directly affected by lead. It only becomes affected through the falling blood pressure. One of the noticeable consequences of respiration continuing is to set in motion the movements of the heart. There is no uniformity in regard to the amount of lead required to cause death. In this circumstance lies an explanation of the idiosyncrasy of certain persons to lead poisoning. Some are more readily influenced than others. In these experiments any difference as regards sex was not apparent, nor had atropin the slightest effect in altering the influence of lead upon the heart. At the autopsies the intestine was not found to be contracted as in the case when lead is administered by the mouth over a period.

In lead poisoning there may be few, if any, naked eye structural alterations found after death, none, at any rate, that can be regarded as pathognomonic. The presence of contracted kidneys, slight œdema of brain, and of albumen in the urine removed from the bladder would, with the history of a patient having had convulsions, suggest uræmia, while these with, in addition, a blue line on the gums, and the history or not of the individual having worked amongst lead, would raise the question of plumbism. The association of lead poisoning and interstitial nephritis is so usual that when after death the kidneys are found contracted, and the occupation followed has been such as to favour the possibility of plumbism, there is a disposition to regard lead as having been the cause of death, and yet other causes than plumbism may have been in operation in regard to which it is difficult to apportion their proper share. Lead workers are not less predisposed, for example, to alcoholism than other persons. In all doubtful cases, and especially in view of the application of the Workmen's Compensation Act, no diagnosis can be complete without a chemical examination of the internal organs and of the urine by a competent analyst. Lead, as already stated, can remain in the system for years. It is excreted, too, long after it has ceased to enter the body. It is difficult to say how far lead, when found in extremely minute quantities in the internal organs, is to be regarded as the cause of death. In acutely fatal cases of saturnine poisoning only the merest traces of lead may be found in the brain, liver, and kidneys, these organs being otherwise healthy, and yet we cannot but attach considerable importance to this small quantity, for in the fatal case above reported only three parts of lead per million of brain tissue were found. Besides, in chronic cases the long-continued passage through the system of minute quantities of lead comes to be followed by structural changes in the internal organs, the presence of which, along with the finding of the merest traces of lead, would be strongly presumptive evidence of plumbism. In cases where there is the possibility of a claim being raised in a law court it is well to have a chemical examination of the internal organs for lead, since the poison is often long retained in the system. In the event of no lead being found in the body of a lead worker wherein pathological changes in kidneys and blood vessels are well marked, the presence of interstitial nephritis would be of some importance. Allusion has been made to the possible part played by alcohol in determining kidney lesions. Probably the influence is secondary, and not direct. The incidence of alcohol is rather upon the liver than upon the kidneys. Even if we admit that painters as a class are as intemperate as other workmen in regard to the use of alcohol, the fact remains that in them interstitial nephritis is met with from four to five times more frequently than in most other workmen, a circumstance which of itself suggests that some occupation influence has been in operation.

It is not my intention to deal with the medicinal treatment of lead poisoning, but I cannot dismiss this subject without referring (1) to the researches of Dr. Jacques Carles, of Bordeaux, upon the important rôle of leucocytes in the absorption and elimination of lead from the body, and (2) to the ionisation of lead by electricity. By causing abscesses Dr. Carles * finds that considerable quantities of lead in the pus can be thrown out from the body, and that this forms an effective method of treating plumbism. Having fed dogs with lead for a few days, he injects into the subcutaneous tissues of the dorso-lumbar region essence of turpentine. A sharp reaction follows, so that within seven days afterwards an abscess forms, from which several grammes of pus can be removed by incision. After destroying the animal under chloroform quantities of the organs equal in weight to that of the pus removed are submitted to chemical analysis. In one dog thus treated Carles found in 26 grammes (*a*) of blood a complete absence of lead, (*b*) in a similar quantity of brain the merest trace of lead, (*c*) in the intestine a visible but imponderable quantity of lead, (*d*) in the liver 0.003 gramme of lead, and in (*e*) the pus from the abscess 0.005 gramme of lead. Although the liver is usually regarded as the principal organ of elimination of lead, the pus from the artificially induced abscess contains more. The absence of lead from the blood is interesting, since it suggests that the leucocytes, which are the carriers, only traverse the circulation, pass out into the tissues

* *Bulletin Général de Therapeutique*, February 8, 1909, p. 161.

and organs, and therein either deposit the lead or themselves become for the time being stationary cells. In other experiments as much as 0.08 gramme of sulphide of lead was obtained from the pus. Carles found after opening the abscesses and removing the pus that lead-poisoned dogs which had suffered from acute colic and constipation lost all their symptoms and quickly recovered. In dogs the subjects of chronic plumbism, and to whom no lead had been given for two months prior to the injection of turpentine, the pus from the abscess contained more lead than any of the internal organs. In some instances the blood contained traces of lead, a circumstance which shows that lead which has been stored up in the tissues can repass into the circulation, and thus explain those unexpected outbursts of acute lead poisoning in patients who had recovered, also cases of chronic plumbism without fresh exposure. Carles tried the treatment upon a painter who was suffering from tremor and incomplete loss of power in his hands. In the pus removed from his artificially induced abscess 2½ milligrams of sulphide of lead were found, although the man had not been near lead for three months. In other patients similar results were obtained. Of all the means adopted for the elimination of lead the production of an "abscess of fixation" is not the least important. According to the Bordeaux physician pus always contains, weight for weight, more lead than do the viscera, and even in old-standing cases the formation of an abscess still has the remarkable power of attracting and locating the poison. Although a somewhat painful method of treatment, there are circumstances under which it might be well to adopt it.

Medical ionisation is of comparatively speaking recent date. When serving on the Dangerous Trades Committee of the Home Office my attention was drawn to the electrical-bath treatment of workmen affected by plumbism, and from whose bodies, when immersed in whole or in part, lead was said to be removed, since the presence of the metal could be chemically proved in the water of the bath and on the submerged metallic rod. As at the time of which I speak I had had no experience of the electric bath, I could offer no opinion upon its utility as a means of treatment. Since then Stephen Leduc, Finsi, and others have placed medical ionisation on quite a scientific basis. Having administered for several months small doses of acetate of lead to a rabbit, in whose fæces submitted to Mr. T. M. Clague for analysis lead was found, the animal under the influence of anæsthesia was placed in an electric bath, with the object of extracting lead from its tissues. Its forelegs were submerged in water containing a small quantity of potassium acetate, and the hind limbs up to the knees in another basin of distilled water, one pole being in each basin. The animal was kept in the bath for fully half an hour, a current of 14 milliamperes being employed. In the water of the bath and on the negative pole traces of lead were found. The rabbit experienced no inconvenience from the treatment. A month afterwards and later the bath treatment was again tried, for slightly longer periods, and under 19 and 25 milliamperes, the results being that not only was lead found on the negative pole and in the water, but the animal improved in condition. By this line of treatment to which Dr. Jones, of St. Bartholomew's Hospital, has on more than one occasion drawn attention, excellent results can be obtained. For many suggestions in regard to the electrolytic treatment of plumbism and for chemical analysis I am indebted to Mr. Clague.

